# Variability of serum phenytoin levels in critically ill head injured patients in intensive care unit

**DOI:** 10.4103/0972-5229.40946

**Published:** 2008

**Authors:** Lalitha V. Pillai, Narendra Vaidya, A. D. Khade, Saiffuddin Hussainy

**Affiliations:** **From:** Department of Critical Care Medicine, Lokmanya Hospitals, Chinchwad, Pune, India

**Keywords:** head injured, phenytoin level, variability

## Abstract

Patients with large variations in phenytoin levels despite standard doses may prove to become difficult clinical problems. Our study of 34 head injury patients whose serum phenytoin levels were measured on day one and day five following intravenous loading and maintenance dose of phenytoin, showed 38.24% patients, to have therapeutic phenytoin levels on day one, while 20% were in toxic range. On day five, 23% patients were in toxic and 29.41% were in therapeutic range. Only 21% patients remained in the therapeutic range during the monitoring period. This study shows that there is a wide variability of phenytoin levels in the ICU patients with a difference of more than 100% between the highest and lowest phenytoin level in individual cases (in four patients the difference exceeded 500%) raising concern about the safety of the drug. Hence it is recommended that intensive care unit patients receiving phenytoin therapy should have periodic serum phenytoin obtained even in absence of seizures or classic signs phenytoin toxicity.

## Introduction

Phenytoin is a widely prescribed anticonvulsant drug in the intensive care unit. For maximum effectiveness this medication needs to be maintained in steady levels in the body. Therapeutic levels are usually between 10-20 mcg/ml. Intravenous loading, oral loading and starting/restarting oral maintenance-dosing can achieve a serum phenytoin level >10 mcg/ml. Stable phenytoin levels at steady state are obtained in most patients seven to ten days after initiation of oral therapy with recommended doses of 300 mg/day. Following rapid intravenous (I.V) infusion of phenytoin, therapeutic serum levels were achieved within 5 to 30 min with a half-life of 31.2 ± 8.4 h.[1]

Variability in serum phenytoin and wide inter patient variability have also been noted in various studies of different population groups.[2–5] Critically ill patients with multiple drug prescriptions and organ dysfunction can have unpredictable drug metabolism. Hence we decided to study the phenytoin levels following intravenous loading dose in head injured patients to study the quantum of variability in the serum phenytoin levels in this group of patients.

### Aim

The aim of the study was to assess the variability of phenytoin levels following a loading dose in critically ill head injured patients.

## Materials and Methods

**Study Design:** ICU Patients ≥18 years of age with moderate to severe head injuries, were selected for this prospective study. Phenytoin was given in a loading dose of 15-20 mg/kg IV at the rate of 50 mg/min, with a maintenance intravenous dose of 300 mg per day in divided doses. Serum phenytoin was measured on the first day and fifth day by (Clia Immunolite Fully Automated Immuno Assay System, Dpc, Usa).

## Results

In all, 34 patients serum phenytoin levels, were measured on the first and the fifth day following loading and maintenance dose of phenytoin. In addition, among these 12 patients phenytoin level were done on day 10^th^ also. There were 29 males and 5 females all of whom had moderate to severe head injuries. The median serum phenytoin level was 11.45 mcg/ml (2.5-44.28 mcg/ml) on the first day and 10.85 mcg/ml (2.4-41 mcg/ml) on the 5^th^ day. There was no statistically significant difference in the median serum phenytoin level from day 1 to day 5 (*P* value = 0.9240). Out of the 34 patients only 21% patients remained in the therapeutic range during the monitoring period.

When individual serum phenytoin levels were studied, [Table 1] 41.18% had sub therapeutic, 38.24% had therapeutic and 20.59% had toxic levels on day one. Five days later 47.06% had sub therapeutic, 29.41% had therapeutic and 23.53% had toxic levels. Table 2 shows the serum phenytoin levels on day five. The pharmacokinetics in twelve patients on day ten is shown in Table 3 and Table 4.

**Table 1:** Serum phenytoin level on day 1 and day 5

**S. Phenytoin level micro gram/ml**	**Day 1**	**%**	**Day 5**	**%**
<10	14	41.18	16	47.06
10.01 to 20	13	38.24	10	29.41
>20	7	20.59	8	23.53
Total	34	100.00	34	100.00

**Table 2:** Variability of serum phenytoin concentrations on 1^st^ day and 5^th^ day

**S. Phenytoin level micro gram/ml**	**Day 1**	**S. Phenytoin level micro gram/ml day 5**	**No. of patients**
<10	14 (41.18)	<10	9 (64.29)
		10.01-20	4 (28.57)
		>20	1 (7.14)
		Total	14
10.01-20	13 (38.24)	<10	5 (38.46)
		10.01-20	5 (38.46)
		>20	3 (23.08)
		Total	13
>20	7 (20.59)	<10	2 (28.57)
		10.01-20	1 (14.29)
		>20	4 (57.14)
		Total	7
Total	34		34

Figures in the parentheses are in percentage

**Table 3:** Pharmacokinetics of serum phenytoin levels on day 1 and 5 of the patients according to day 1

**S. Phy. Level (microgram/ml)**	**Day 1**	**S. Phy. Level (microgram/ml)**	**Day 5**	**Day 10**
<10 (Sub-theraupetic)	8 (66.67)	<10	3 (37.5)	3 (37.5)
		10.01-20.00	4 (50)	3 (37.5)
		20.01 and above	1 (12.5)	2 (25)
		Total	8	8
10.01-20.00	3 (25)	<10	1 (33.34)	2 (66.67)
		10.01-20.00	1 (33.34)	0
		20.01 and above	1 (33.34)	1 (33.33)
		Total	3	3
20.00 and above	1 (8.34)	<10	1 (100)	1 (100)
		10.01-20.00	0	0
		20.01 and above	0	0
		Total	1	1

Figures in the parentheses are in percentage

**Table 4:** Phenytoin level day 1, day 5 and day 10 [12 patients]

**S. Phenytoin level micro gram/ml**	**Day 1**	**%**	**Day 5**	**%**	**Day 10**	**%**
2.4 to 10.00	8	66.67	5	41.67	6	50
10.01 to 20	3	25.00	5	41.67	3	25
20.01 and above	1	8.33	2	16.67	3	25
Total	12	100.00	12	100.00	12	100

Patients with toxic levels on day one and day five had a median age of 55 years (SD ± 18.39) and 49 (SD ± 21.60) years respectively There were three patients who had toxic level on day one and therapeutic or sub-therapeutic by day 5.They had median age of 55 years, while patients with normal or sub therapeutic levels on day one and day five had median age of 38 (SD ± 21.03) years and 39 (SD ± 20.25) years respectively.

Liver function tests were available for 20 of these patients and were abnormal in ten patients. But there was no correlation between the toxic levels and raised transaminases nor did low albumin levels relate to sub therapeutic level. In patients with serum phenytoin levels in toxic range on day one, the average Serum Glutamic Oxaloacetic Transaminase (SGOT) and Serum Glutamic Pyruvic Transaminase (SGPT) was 65.4 IU/L and 76.2 IU/L as against 108 IU/L and 55.5 IU/L for those with subtherapeutic and normal levels. On day five, the average SGOT and SGPT was 100.8 IU/L and 52.8 IU/L for patients with toxic levels vs. 49 IU/L and 65.4 IU/L for normal and sub therapeutic levels. Albumin level was 3.75 gm/dl for those with sub therapeutic level vs. 3.63 gm/dl for those with normal or toxic level.

## Discussion

This study shows the wide variability of serum phenytoin levels after recommended parental loading and maintenance dose of phenytoin in the critically ill head injured patients in the intensive care unit raising concern about the safety of the drug.

Unbound fraction of phenytoin is the therapeutically active drug, but routinely the total plasma concentration is monitored and used as surrogate in dose adjustment regimes and hence total plasma concentration was used in our study. Phenytoin is highly protein bound but many factors other than serum albumin levels influence the drug levels of phenytoin. The liver being the chief site of biotransformation of phenytoin, seriously ill patients with impaired liver function and elderly patients, may show early signs of toxicity. Although toxic levels in our study, both on the first and on the fifth day occurred in patients older than those with normal or sub therapeutic level, it was not statistically significant probably because numbers in individual groups were small.

Markowesky *et al.*, in their study concluded that phenytoin protein binding significantly correlated with albumin and was more variable in ICU and convalescent patients with brain injuries than in healthy volunteers.[6] In our study, neither albumin nor transaminse levels appeared to correlate with raised or sub therapeutic phenytoin levels. In their study the phenytoin requirement decreased during convalescent. Our study did not include convalescent patients but even when studied up to 10 days there were unpredictable fluctuations in the levels.

Theodore and colleagues reported a pseudo steady state like phenomenon lasting for 5 to 10 days with plasma phenytoin levels remaining stable for 2 to 12 days after a dosage change. Subsequently, the levels fluctuated by more than 25% in the next 5 to 22 days and a final steady-state level was reached between 13 to 31 days after the first dosage change. In our study steady state therapeutic level was seen to occur only in 21% of cases while in the rest, there was a wide fluctuation in the levels with a difference of more than 100% between the highest and lowest phenytoin level in individual cases (in four patients the difference exceeds 500%).

These fluctuations in the phenytoin levels have been explained by the changes in the metabolism of the drug in critically ill patients in various studies.[6–8] Boucher *et al.*, correlated fall in phenytoin levels to increase in urinary clearance, which they postulated to be a consequence of changes in protein binding, induction of metabolism or the influence of stress on hepatic metabolic capacity. Martinnelli *et al.*,[8] have suggested the necessity to individualize dosing after the fifth day following rapid intravenous loading to maintain safe and therapeutic levels. Bayesian regression programme has been used to forecast an estimate of each subject's individual pharmacokinetics[9] in various studies.

## Conclusion

Only 38.24% patients, had therapeutics phenytoin level on day one, while 20% were in toxic range. On day five, 23% patients were in toxic and 29.41% were in therapeutic range. This study shows that there is a wide variability of phenytoin levels in the ICU patients with a difference of more than 100% between the highest and lowest phenytoin level in individual cases (In four patients the difference exceeds 500%). Potential mechanisms for this include the influences of poly pharmacy, altered drug metabolism, acid/base status and other factors beyond serum albumin levels. These results raise concern about the safety of phenytoin doses. Hence it is recommended that ICU patients receiving phenytoin therapy should have periodic serum phenytoin obtained even in absence of seizures or classic signs of phenytoin toxicity.
